# Landscape of HER2-low metastatic breast cancer (MBC): results from the Austrian AGMT_MBC-Registry

**DOI:** 10.1186/s13058-021-01492-x

**Published:** 2021-12-14

**Authors:** Simon Peter Gampenrieder, Gabriel Rinnerthaler, Christoph Tinchon, Andreas Petzer, Marija Balic, Sonja Heibl, Clemens Schmitt, August Felix Zabernigg, Daniel Egle, Margit Sandholzer, Christian Fridolin Singer, Florian Roitner, Christopher Hager, Johannes Andel, Michael Hubalek, Michael Knauer, Richard Greil

**Affiliations:** 1 Department of Internal Medicine III With Haematology, Medical Oncology, Haemostaseology, Infectiology and Rheumatology, Oncologic Center, Paracelsus Medical University Salzburg, Müllner Hauptstraße 48, 5020 Salzburg, Austria; 2 Laboratory for Immunological and Molecular Cancer Research (LIMCR) and Center for Clinical Cancer and Immunology Trials (CCCIT), Salzburg Cancer Research Institute (SCRI), Salzburg, Austria; 3 Cancer Cluster Salzburg, Salzburg, Austria; 4 Internal Medicine - Department for Haemato-Oncology, LKH Hochsteiermark-Leoben, Leoben, Austria; 5 Internal Medicine I for Hematology With Stem Cell Transplantation, Hemostaseology and Medical Oncology, Ordensklinikum Linz Barmherzige Schwestern – Elisabethinen, Linz, Austria; 6 Division of Oncology, Department for Internal Medicine, Medical University Graz, Graz, Austria; 7 Department of Internal Medicine IV, Klinikum Wels-Grieskirchen GmbH, Wels, Austria; 8 Department of Hematology and Internal Oncology, Kepler University Hospital, Johannes Kepler University Linz, Linz, Austria; 9 Department of Internal Medicine, County Hospital Kufstein, Kufstein, Austria; 10 Department of Gynaecology, Medical University Innsbruck, Innsbruck, Austria; 11 Department of Internal Medicine II, Academic Teaching Hospital Feldkirch, Feldkirch, Austria; 12 Department of Obstetrics and Gynecology and Comprehensive Cancer Center, Medical University of Vienna, Vienna, Austria; 13 Department of Internal Medicine II, Hospital Braunau, Braunau, Austria; 14 Breast Center Dornbirn, Dornbirn, Austria; 15 Department of Internal Medicine II, Pyrn-Eisenwurzen Klinikum Steyr, Steyr, Austria; 16 Department of Gynecology, Breast Health Center Schwaz, Schwaz, Austria; 17 Breast Center Eastern Switzerland, St. Gallen, Switzerland

**Keywords:** Metastatic breast cancer, HER2-low, HER2-positive, HER2-negative, OS, PFS, Registry, Real-world data

## Abstract

**Background:**

About 50% of all primary breast cancers show a low-level expression of HER2 (HER2-low), defined as immunohistochemically 1+ or 2+ and lack of HER2 gene amplification measured by in situ hybridization. This low HER2 expression is a promising new target for antibody–drug conjugates (ADCs) currently under investigation. Until now, little is known about the frequency and the prognostic value of low HER2-expression in metastatic breast cancer (MBC).

**Patients and methods:**

The MBC-Registry of the Austrian Study Group of Medical Tumor Therapy (AGMT) is a multicenter nationwide ongoing registry for MBC patients in Austria. Unadjusted, univariate survival probabilities of progression-free survival (PFS) and overall survival (OS) were calculated by the Kaplan–Meier method and compared by the log-rank test. Multivariable adjusted hazard ratios were estimated by Cox regression models. In this analysis, only patients with known HER2 status and available survival data were included.

**Results:**

As of 11/15/2020, 1,973 patients were included in the AGMT-MBC-Registry. Out of 1,729 evaluable patients, 351 (20.3%) were HER2-positive, 608 (35.2%) were HER2-low and 770 (44.5%) were completely HER2-negative (HER2-0). Low HER2-expression was markedly more frequent in the hormone-receptor(HR)+ subgroup compared to the triple-negative subgroup (40% vs. 23%). In multivariable analysis, low HER2 expression did not significantly influence OS neither in the HR+ (HR 0.89; 95% CI 0.74–1.05; *P* = 0.171) nor in the triple-negative subgroup (HR 0.92; 95% CI 0.68–1.25; *P* = 0.585), when compared to completely HER2-negative disease. Similar results were observed when HER2 IHC 2+ patients were compared to IHC 1+ or 0 patients.

**Conclusion:**

Low-HER2 expression did not have any impact on prognosis of metastatic breast cancer in this real-world population.

**Supplementary Information:**

The online version contains supplementary material available at 10.1186/s13058-021-01492-x.

## Introduction

Amplification of human epidermal growth factor receptor 2 (HER2) is a well-established negative prognostic factor both in early and metastatic breast cancer (MBC). HER2-directed therapies, however, have changed the natural course of this disease. Nowadays, adequately treated HER2+ /hormone-receptor(HR)+ breast cancer belongs to the subtypes with the most favorable prognosis both in the early and the advanced stage [1, 2].

In contrast to HER2 positivity, defined as immunohistochemically (IHC) 3+ or IHC 2+ and HER2 gene amplification measured by in situ hybridization (ISH) [3], the significance of a low-level expression of HER2 (HER2-low) is less clear. HER2-low is defined as IHC 1+ or IHC 2+ without HER2 gene amplification and compromises about 50 to 55% of all primary breast cancers [3, 4]. In general, these tumors do not respond to trastuzumab [5] or T-DM1 [6], even if there seems to be a subgroup of patients—selected by a novel poly-ligand profiling technique—who might benefit from trastuzumab [7]. HER2-low, however, is a potential target of new antibody–drug conjugates (ADCs). In contrast to T-DM1, these new ADCs show a higher bystander killer effect, by using cleavable linkers and a higher drug-to-antibody ratio [8, 9] and are therefore not only active in HER2-overexpressing tumors [10] but also in tumors with low HER2 expression. Two of these ADCs have already shown promising activity in phase I trials including HER2-low MBC [11, 12]: trastuzumab deruxtecan and trastuzumab duocarmazine. The former ADC is already approved by the US Food and Drug Administration (FDA) and the European Medicines Agency (EMA) for the treatment of HER2+ MBC pretreated with two or more anti-HER2-based regimens. For the treatment of HER2-low MBC, however, none of the ADCs is approved today. Currently, two phase III trials are investigating trastuzumab deruxtecan in patients with HER2-low MBC (DESTINY-Breast04; ClinicalTrials.gov identifier: NCT03734029 and DB-06; NCT04494425). Furthermore, another novel HER2-targeting ADC (RC-48) is tested in HER2-low MBC in a phase I/II trial (NCT03052634). Besides several ADCs, bispecific antibodies as well as HER2 vaccines are under investigation in HER2-low breast cancer as reviewed by Tarantino et al. [13].

Several retrospective studies investigated the prognostic value of low HER2 expression in early breast cancer (EBC) [14–20]. Most of these studies showed a negative impact of a IHC 2+ HER2-expression on the risk of recurrence or survival but no or little influence on outcome by HER2 IHC 1+. In contrast to EBC, little is known about the real frequency and the prognostic significance of this new breast cancer subtypes in MBC. Only a few retrospective studies have currently been published, which investigated the prognostic value of low HER2 expression in MBC [20–22]. One trial showed a negative prognostic value of HER2 IHC 2+ only in patients older than 55 years, while there was no OS difference between IHC 2+ and 0 or 1+ tumors in the overall population [20]. The other two publications did not find any difference in OS between patients with HER2-low tumors compared to patients with completely HER2-negative tumors irrespective of the hormone receptor status [21, 22], however the patient numbers in these studies were rather low.

Here, we present data from a large nation-wide registry for MBC in Austria. Both the incidence and the prognostic value of low HER2 expression were investigated in dependence of the HR status.

## Methods

The MBC-registry of the Austrian Study Group of Medical Tumor Therapy (AGMT) is a multicenter nationwide ongoing retrospective and prospective registry for MBC patients in Austria.

HER2+ was defined as IHC 3+ or IHC 2+ and ISH+ according to the American Society of Clinical Oncology/College of American Pathologists Clinical Practice (ASCO/CAP) guidelines [3]. HER2-low was defined as IHC 1+ or IHC 2+ and ISH-. Completely, HER2-negative (HER2-0) was defined as IHC 0 and ISH- (if available). The classification was based on local pathology reports. No central pathology review was performed.

In this analysis, only patients with known HER2 status and available survival data were included. For progression-free survival (PFS) analyses, only patients with at least one line of therapy for metastatic disease and sufficiently documented medical records allowing calculation of PFS were included.

In patients with more than one available tumor sample, the following hierarchy was applied: if a tumor sample from a metastatic site was accessible, which was taken within 3 months after diagnosis of metastatic disease and included at least ER- and HER2-status, the receptor status (as well as grade, Ki-67 and histologic subtype) of this biopsy was used. Otherwise, the receptor status of the latest primary tumor (or local recurrence) diagnosed before (or within 3 months of) the diagnosis of metastatic disease was used.

The primary goal of this analysis was to determine the frequency of HER2+, HER2-low and HER2-0 in this MBC population in dependency of the HR status. Furthermore, the impact of low HER2-expression on overall survival (OS) in the HR+ and triple negative population was investigated, respectively. Primarily, HER2-low was compared with HER2-0 regarding OS and first-line PFS, both in univariate and in multivariable analysis in the HR+ and triple-negative population, respectively. Secondarily, IHC 2+ was compared to IHC 1+ or IHC 0 in the two mentioned populations.

Overall survival was calculated from diagnosis of metastatic disease until death from any cause. First-line PFS was defined as time from start of first-line therapy until progression or death from any cause. In order to prevent falsely long PFS times in this retrospective analysis, patients who died more than two month after the end of therapy were censored with date of last dose.

Unadjusted and univariate PFS and OS probabilities between subgroups and were compared by the log-rank test. Multivariable adjusted hazard ratios (HR) were estimated by Cox proportional hazards models. Multivariable analysis was performed separately for HR+ and HR- subgroups with HER2-status (low vs. 0), disease-free survival (DFS; ≥ 24 months or de novo metastatic vs. < 24 months) and visceral disease (yes vs. no) as minimum model. For inclusion of age (continuous), menopausal status (premenopausal vs. postmenopausal) and number of metastatic sites (2–3 vs. 1 and ≥ 4 vs. 1) stepwise backward selection according to Akaike’s information criterion (AIC) were performed. As a result of the algorithm (Additional file 1: Table S5–S8), all variables were included in the final models. Subsequently model stability investigations were performed according to Heinze G et al. [14]. Due to nonlinear influence of age on survival, age was finally included according to menopausal status (interaction).

All tests were carried out at the 5% significance level, no p-value correction was applied. All statistical analyses were performed using R (version 4.0.2). Important packages: survminer (for survival analysis), bootStepAIC (for variable selection).

## Results

### Frequency of low HER2 expression

**Fig. 1:**
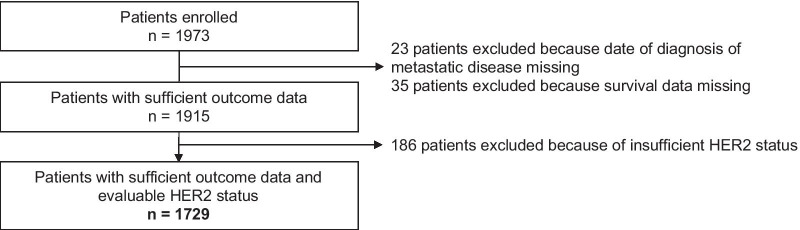
Consort diagram

**Fig. 2:**
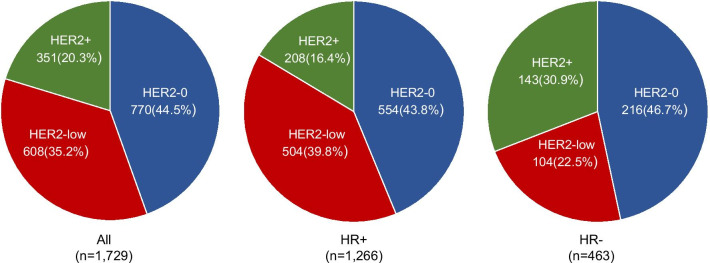
Frequencies of the different expression levels of HER2 in dependency of the hormone receptor (HR) status. HER2+  =  HER2-positive (immunohistochemically [IHC] 3+ or IHC 2+ and ISH+); HER2-low = low HER2 expression (IHC 1 or IHC 2+ and ISH−); HER2-0 = completely HER2-negative (IHC 0)

As of 11/15/2020, 1,973 patients were included in the AGMT-MBC-Registry (Fig. 1). Out of 1,729 evaluable patients, who were diagnosed with MBC between November 2000 and August 2020, 351 (20.3%) were HER2-positive, 608 (35.2%) were HER2-low and 770 (44.5%) were completely HER2-negative (HER2-0) (Fig. 2). In 459 patients (26.5%), the receptor status was determined in metastatic tissue and in 1270 patients (73.5%) in the primary tumor. Low HER2-expression was markedly more frequent in the HR+ subgroup compared to the HR- (triple-negative) subgroup (40% vs. 23%). The frequencies of all three HER2 subgroups in dependency of the HR status are shown in Fig. 2. When HER2patients were excluded, 44% of all HER2-negative patients, 48% of patients with HR+ /HER2- tumors and 33% of patients with triple-negative tumors showed a low-level expression of HER2, respectively.

Compared to HER2-0 patients, patients with HER2-low tumors were significantly older, were significantly more frequent de novo metastatic, HR+ and of no special type (NST) histology, respectively. Detailed patient characteristics for the overall population as well as for the three different expression levels of HER2 are provided in Table 1.

**Table 1:** Patient characteristics

	All (*n* = 1729) N (%)		HER2-0 (*n* = 770) N (%)		HER2-low (*n* = 608) N (%)		HER2+ (*n* = 351) N (%)		P HER2-low versus HER2-0
Median age (range)*	63 (24–97)		62 (27–97)		64 (26–95)		61 (24–94)		**0.017**^‡^
*Stage at initial diagnosis*									
Stage I–III	1119	(64.7)	536	(69.6)	388	(63.8)	195	(55.6)	**0.066**
DFS < 24 months	366	(32.7)	179	(33.4)	119	(30.7)	68	(34.9)	
DFS ≥ 24 months	696	(62.2)	328	(61.2)	253	(65.2)	115	(59)	
DFS NA**	57	(5.1)	29	(5.4)	16	(4.1)	12	(6.2)	
De novo metastatic	602	(34.8)	231	(30.0)	217	(35.7)	154	(43.9)	
Unknown	8	(0.5)	3	(0.4)	3	(0.5)	2	(0.6)	
Menopausal status*									
Postmenopausal	1148	(66.4)	501	(65.1)	429	(70.6)	218	(62.1)	**0.029**
Premenopausal	226	(13.1)	108	(14.0)	60	(9.9)	58	(16.5)	
Unknown	336	(19.4)	154	(20.0)	109	(17.9)	73	(20.8)	
Male***	19	(1.1)	7	(0.9)	10	(1.6)	2	(0.6)	
Metastatic sites*									
Visceral disease	836	(48.4)	337	(43.8)	279	(45.9)	220	(62.7)	0.464
Non-visceral disease only	893	(51.6)	433	(56.2)	329	(54.1)	131	(37.3)	
Number of metastatic sites*									
Median (range)	1	(1–9)	1	(1–6)	1	(1–8)	1	(1–9)	0.793
1	951	(55)	432	(56.1)	332	(54.6)	187	(53.3)	
2–3	661	(38.2)	288	(37.4)	232	(38.2)	141	(40.2)	
≥ 4	117	(6.8)	50	(6.5)	44	(7.2)	23	(6.6)	
*Hormone-receptor (HR) status*									
Positive	1266	(73.2)	554	(71.9)	504	(82.9)	208	(59.3)	**< 0.001**
Negative	463	(26.8)	216	(28.1)	104	(17.1)	143	(40.7)	
*Histologic subtype*									
No special type (NST)	1205	(69.7)	503	(65.3)	433	(71.2)	269	(76.6)	**0.010**
Invasive lobular	228	(13.2)	129	(16.8)	80	(13.2)	19	(5.4)	
Other	224	(13.0)	101	(13.1)	82	(13.5)	41	(11.7)	
Unknown	72	(4.2)	37	(4.8)	13	(2.1)	22	(6.3)	
*Grade*									
1	70	(4.0)	36	(4.7)	29	(4.8)	5	(1.4)	0.032
2	751	(43.4)	314	(40.8)	295	(48.5)	142	(40.5)	
3	645	(37.3)	293	(38.1)	202	(33.2)	150	(42.7)	
Unknown	263	(15.2)	127	(16.5)	82	(13.5)	54	(15.4)	
*Treatment for early stage*									
(Neo)adj. chemotherapy	705	(63.0)	337	(62.9)	226	(58.2)	142	(72.8)	0.176
(Neo)adj. trastuzumab ± pertuzumab	133	(11.9)	13	(2.4)	17	(4.4)	103^†^	(52.8)^†^	0.142
Adj. endocrine therapy (HR+ only)	623	(78.9)	299	(79.7)	245	(79.8)	79	(73.1)	1.000
*Treatment for metastatic disease*									
Chemotherapy	1161	(67.1)	499	(64.8)	378	(62.2)	284	(80.9)	0.341
Trastuzumab	315	(18.2)	12	(1.6)	29	(4.8)	274	(78.1)	**0.001**
Pertuzumab	170	(9.8)	5	(0.6)	20	(3.3)	145	(41.3)	**0.001**
T-DM1	105	(6.1)	5	(0.6)	8	(1.3)	92	(26.2)	0.322
Lapatinib	119	(6.9)	3	(0.4)	9	(1.5)	107	(30.5)	0.061
Endocrine therapy (HR+ only)	1033	(81.6)	465	(83.9)	429	(85.1)	139	(66.8	0.655
CDK4/6 inhibitor (HR+ only)	443	(35.0)	213	(38.4)	215	(42.7)	15	(7.2)	0.183

Statistically significant *p*-values are highlighted in bold

*At diagnosis of metastatic disease

**No surgery or incomplete surgery date

***Not included in subgroup comparison with chi-square tests

^†^17 patients did not receive any (neo-)adjuvant therapy, 11 patients were diagnosed before trastuzumab was available, 20 patients had HER2- primary tumors but HER2+ metastatic disease

^‡^Calculated with Wilcoxon test. All other *p*-values result from Chi-square tests

### Impact of low HER2 expression on OS

Patients with HER2+ MBC had a significantly better prognosis compared to both patients with HER2-low and HER2-0 tumors (median OS 38.3 vs. 34.2 vs. 26.8 months; HR 0.69; 95% CI 0.59–0.81; *P* < 0.001 and HR 0.84; 95% CI 0.73–0.95; *P* = 0.006) months respectively; Additional file 1: Fig. S1).

**Fig. 3:**
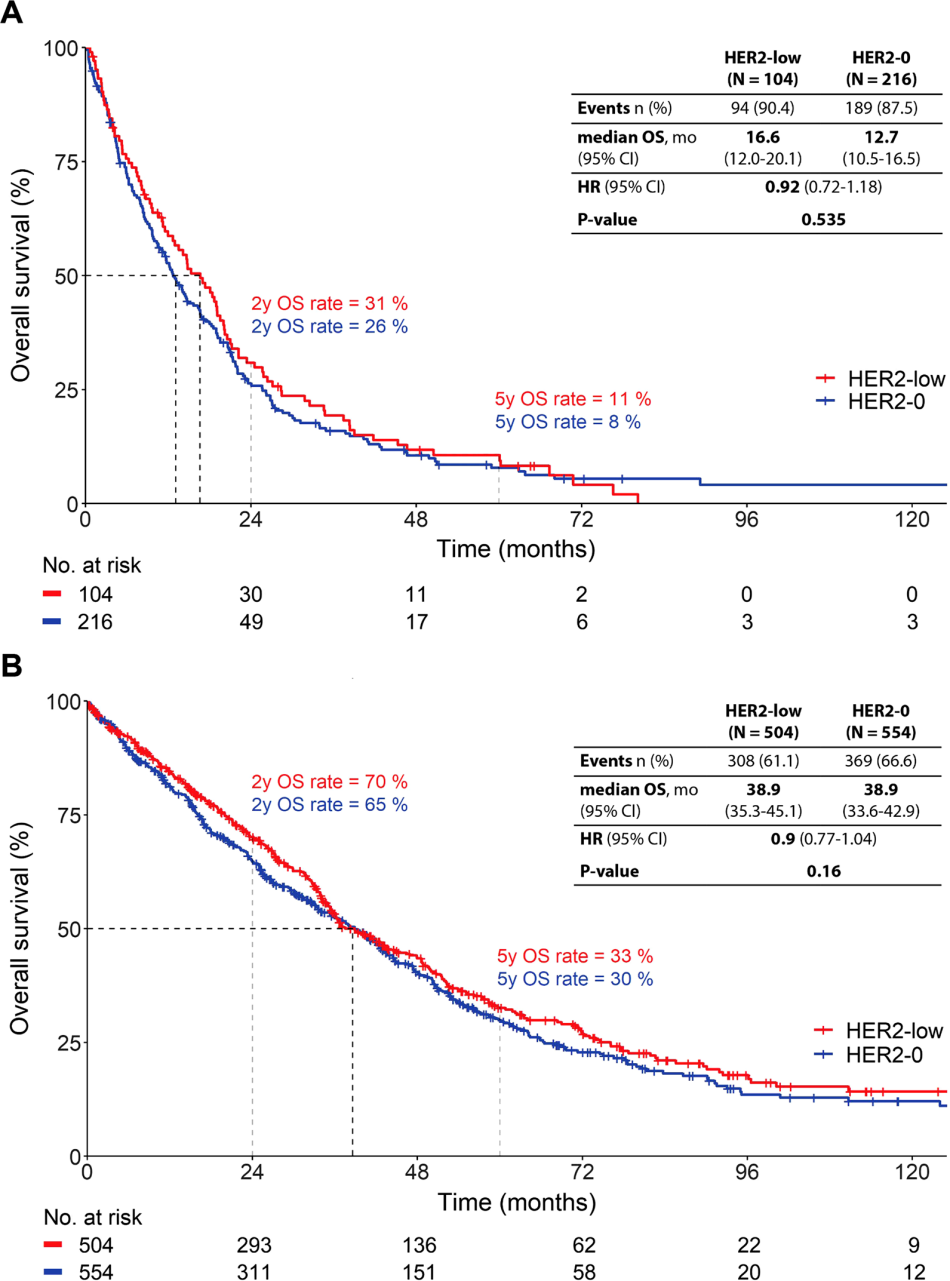
OS of patients with HER2-low tumors and patients with completely HER2-negative tumors (HER2-0) in (**A**) the HR-negative [*n* = 320] and (**B**) the HR-positive subgroup [*n* = 1058], respectively

In this analysis, we focused on the prognostic differences between the HER2-low and HER2-0 subgroup. The median follow-up in this population was 68.2 months (95% CI 61.6–72.3 months). In univariate analysis, HER2-low was significantly associated with a longer OS compared to completely HER2-negative disease (HR 0.84; 95% CI 0.73–0.95; *P* = 0.006; Additional file 1: Fig. S2). Given the unalterable influence of the HR-status on prognosis and the uneven distribution of HR-positivity between the two HER2-subgroups, all further analyses were performed in HR+ and HR- patients separately. In the triple-negative subgroup, median OS was 16.6 months in HER2-low patients and 12.7 months in HER2-0 patients (HR 0.92; 95% CI 0.72–1.18; *P* = 0.535; Fig. 3A). In the HR+ subgroup, the median OS was 38.9 months both in the HER2-low and in the HER2-0 subgroup (HR 0.90; 95% CI 0.77–1.04, *P* = 0.160; Fig. 3B).

Similarly, in multivariable analysis including the known prognostic factors age (according to menopausal status), duration of disease-free survival and presence of visceral disease and metastatic sites at diagnosis of metastatic disease, we did not observe a statistically significant difference between patients with HER2-low and HER2-0 tumors both the HR+ (HR 0.89; 95% CI 0.74–1.05; *P* = 0.171; Table 2) and in the triple-negative subgroup (HR 0.92; 95% CI 0.68–1.25; *P* = 0.585; Table 3).

**Table 2:** Multivariable analysis (Cox proportional hazard model) of OS for HR+ MBC

*N* = 832 (events 525)	HR	95% CI	*P*
*Age (continuous) according to menopausal status*			
Premenopausal	1.04	1.03–1.06	** < 0.001**
Postmenopausal	1.03	1.02–1.04	** < 0.001**
*DFS*			
≥ 24 months or de novo versus < 24 months	0.75	0.59–0.95	**0.017**
Visceral versus no visceral disease*	1.26	1.02–1.55	**0.030**
Number of metastatic sites*			
2–3 versus 1	1.25	1.02–1.53	**0.029**
≥ 4 versus 1	1.73	1.16–2.59	**0.007**
HER2-low versus HER2-0	0.89	0.74–1.05	0.171

Statistically significant p-values are highlighted in bold

*At diagnosis of metastatic disease

**Table 3:** Multivariable analysis (Cox proportional hazard model) of OS for HR-negative MBC

*N* = 227 (events 199)	HR	95% CI	*P*
*Age (continuous) according to menopausal status*			
Premenopausal	1.04	1.01–1.06	**0.004**
Postmenopausal	1.02	1.01–1.04	**0.002**
*DFS*			
≥ 24 months or de novo versus < 24 months	0.59	0.44–0.80	**0.001**
Visceral versus no visceral disease*	1.59	1.15–2.20	**0.005**
Number of metastatic sites*			
2–3 versus 1	1.23	0.89–1.68	0.210
≥ 4 versus 1	2.01	1.17–3.48	**0.012**
HER2-low versus HER2-0	0.92	0.68–1.25	0.585

Statistically significant p-values are highlighted in bold

*At diagnosis of metastatic disease

### Impact of low HER2 expression on first-line PFS

In univariate analysis, HER2-low did not show a significant influence on PFS when compared to HER2-0: in the HR+ subgroup, median PFS was 15.9 months in the HER2-low subgroup and 13.6 months in HER2-0 subgroup (HR 0.91; 95% CI 0.79−1.05; *P* = 0.189; Additional file 1: Fig. S3). In the triple-negative subgroup, the median PFS was 5.9 months in the HER2-low and 5.5 months in the HER2-0 subgroup (HR 0.93; 95% CI 0.71−1.21; *P* = 0.590; Additional file 1: Fig. S4).

Similar to the OS analysis, in multivariable analysis, we did not find a statistically significant difference between the first-line PFS of patients with HER2-low and HER2-0 tumors both the HR+ (HR 0.92; 95% CI 0.78–1.08; *P* = 0.308; Additional file 1: Table S1) and in the triple-negative subgroup (HR 0.98; 95% CI 0.70–1.37; *P* = 0.908; Additional file 1: Table S2).

### Comparison of HER2 2+ and HER2 1+ /0

As next step, we compared HER2 2+ tumors with HER2 0 or 1+ tumors in the HR+ and triple negative cohort, respectively. Similar to the previous OS analysis, we did not find any prognostic differences between these two HER2-expression groups in the univariate analysis. This was true for the whole HER2-negative cohort (HR 0.99; 95% CI 0.8−1.23; *P* = 0.945; Additional file 1: Fig. S5), the HR+ /HER2- cohort (HR 1.01; 95% CI 0.78−1.31; *P* = 0.957; Additional file 1: Fig. S6) and the triple-negative cohort (HR 0.93; 95% CI 0.63−1.39; *P* = 0.732; Additional file 1: Fig. S7).

## Discussion

Previous studies have shown a negative prognostic impact of HER2 IHC 2+ expression in EBC even in the absence of HER2 amplification [14–20]. Tumor biologic findings in early breast cancer cannot simply be transferred to the metastatic stage, since the genetic background is different between the early and the advanced disease [23] and prognostic factors can behave differently depending on the context. For example, androgen receptor (AR) expression predicted a better prognosis in HR+ EBC [24], while there was no influence on time-to-progression (TTP) on first-line endocrine therapy in MBC [25].

Here we provide, complementary evidence for the incidence of HER2-low in MBC an its influence on prognosis in the HR+ and triple-negative subgroup. Out of the whole MBC cohort (n = 1,729), 35% of patients had HER2-low tumors defined as HER2 IHC 1+ or IHC 2+ and ISH-. This corresponds to 44% of all HER2-negative patients, 48% of patients with HR+/HER2- tumors and 33% of patients with TNBC.

Patients with low HER2 expression did not have a significantly different OS compared to patients without any HER2 expression both in the triple-negative cohort (HR 0.92; 95% CI 0.72–1.18; *P* = 0.535; Fig. 3A) and in the HR+ cohort (HR 0.90; 95% CI 0.77–1.04, *P* = 0.160; Fig. 3B). These results did not chance when HER2 IHC 2+ patients were compared to IHC 1+ or 0 patients.

The large and very detailed database of the AGMT MBC-registry allowed adjusting for important risk factors like age, disease-free survival and location of metastases. Similar to the univariate analyses, in multivariable analysis, the differences in OS between patients with HER2-low and HER2-0 were not statistically significant both in the HR+ (HR 0.89; 95% CI 0.74–1.05; *P* = 0.171) and in the triple-negative subgroup (HR 0.92; 95% CI 0.68–1.25; *P* = 0.585).

The major limitation of our analysis is that the receptor status was extracted from the pathology report and no central HER2 (and ER) testing was performed. The known inter-pathologist variability [26] could have potential impact on our results. Furthermore, the technique of staining and the details of interpretation have slightly changed over time [3]. Since the diagnosis of MBC ranged in a timeframe of 20 years, this is another potential confounder.

Our data are of importance, because new therapeutic options are on the horizon for this new breast cancer subtype. Several antibody–drug conjugates (ADCs), vaccines and bispecific antibodies are currently under development in HER2-low MBC [13]. In a phase Ib study, including 54 extensively pretreated patients with HER2-low MBC (median 7.5 prior therapies), the ADC trastuzumab deruxtecan showed a confirmed objective response rate (ORR) of 37.0% (95% CI 24.3–51.3%) and a median PFS of 11.1 months (95% CI 7.6 months-not evaluable). No difference in activity was seen between tumors with 1+ and 2+ HER2 expression, respectively (ORR 35.7% and 38.5%) [11]. Similarly, trastuzumab duocarmazine showed an ORR of 28% (95% CI 13.8–46.8) in patients with HER2-low/HR+ MBC (n = 32) and of 40% (95% CI 16.3–67.6) in patients with HER2-low/HR- breast cancer in phase I [12].

Currently, two phase III trials are randomizing between trastuzumab deruxtecan and investigator's choice chemotherapy in patients with HER2-low MBC. DESTINY-Breast04 (ClinicalTrials.gov identifier: NCT03734029) includes patients with HR+ and HR- disease pretreated with one or two lines of chemotherapy. DB-06 (NCT04494425), instead, recruits only patients with HR+/HER2-low MBC who have had disease progression on at least two previous lines of endocrine therapies but are naïve for chemotherapy.

Our data are well in line with those from other retrospective analyses, showing no difference in OS between patients with low HER2 expression compared to patients with HER2 0 (or 1+) [20–22]. In one analysis, patients older than 55 years had a statistically significant worse prognosis in case of 2+ HER2 expression compared to HER2 0/1+ patients (HR 1.45; 95% CI 1.01–2.07; *P* = 0.044) [20]. This observation was not confirmed in our study. In an exploratory multivariable analysis, we did not find a statistically significant influence of HER2-low on OS neither in premenopausal (HR 1.10; 95% CI 0.67–1.82; *P* = 0.705; Additional file 1: Table S3) nor in postmenopausal women (HR 0.84; 95% CI 0.70–1.01; *P* = 0.069; Additional file 1: Table S4) with HR+ MBC.

## Conclusion

In our analysis, about 44% of all patients with MBC, defined as HER2-negative according to the ASCO/CAP guidelines [3], showed a low expression of HER2. HER2-low was more frequently in patients with HR+ tumors compared to patients with TNBC (48% vs. 33%). This potentially new target for anti-HER2 ADCs, however, did not show any impact on OS or first-line PFS in this real-world population when HR-expression and other prognostic factors were considered.

## Supplementary Information

**Additional file 1: Figure S1.** OS of patients with HER2-low tumors, patients with completely HER2-negative tumors (HER2-0) and patients with HER2-positive tumors (HER2-pos) in the overall population (n = 1,729). **Figure S2.** OS of patients with HER2-low tumors and patients with completely HER2-negative tumors (HER2-0) in the overall population (n = 1,378). **Figure S3.** PFS of patients with HER2-low tumors and patients with completely HER2-negative tumors (HER2-0) in the HR+ population (n = 961). **Figure S4.** PFS of patients with HER2-low tumors and patients with completely HER2-negative tumors (HER2-0) in the HR-negative population (n = 272). **Figure S5.** OS of patients with HER2 2+ tumors and patients with HER2 0 or 1+ tumors in the overall population (n = 1,378). **Figure S6.** OS of patients with HER2 2+ tumors and patients with HER2 0 or 1+ tumors in the HR+ population (n = 1,058). **Figure S7.** OS of patients with HER2 2+ tumors and patients with HER2 0 or 1+ tumors in the HR-negative population (n = 320). **Table S1.** Multivariate analysis (Cox proportional hazard model) of PFS for HR+ MBC. **Table S2.** Multivariate analysis (Cox proportional hazard model) of PFS for HR-negative MBC. **Table S3.** Multivariate analysis (Cox proportional hazard model) of OS for premenopausal patients with HR+ MBC. **Table S4.** Multivariate analysis (Cox proportional hazard model) of OS for postmenopausal patients with HR+ MBC. **Table S5.** HR+ model stability investigations according Heinze G. et al. [17]. **Table S6.** HR+ model selection frequencies according Heinze G. et al. [17]. **Table S7.** HR- Model stability investigations according Heinze G. et al. [17]. **Table S8.** HR- Model selection frequencies according Heinze G. et al. [17].

## Data Availability

The datasets used and/or analyzed during the current study are available from the corresponding author on reasonable request.

## Abbreviations

ADC: Antibody–drug conjugate
AGMT: Austrian Study Group of Medical Tumor Therapy
AIC: Akaike’s information criterion
ASCO: American Society of Clinical Oncology
CAP: College of American Pathologists
CI: Confidence interval
DFS: Disease-free survival
EBC: Early breast cancer
EMA: European Medicines Agency
FDA: Food and Drug Administration
HER2: Human epidermal growth factor receptor 2
HER3: Human epidermal growth factor receptor 3
HR: Hazard ratio
HR: Hormone receptor
IHC: Immunohistochemically
ISH: In-situ hybridization
MBC: Metastatic breast cancer
NST: No special type
OS: Overall survival
PFS: Progression-free survival
TNBC: Triple negative breast cancer
