# Supplementary material for: Landscape of HER2-low metastatic breast cancer (MBC): results from the Austrian AGMT_MBC-Registry
Source: Breast Cancer Res. 2021 Dec 14;23:112. doi: 10.1186/s13058-021-01492-x (PMC8670265; doi:10.1186/s13058-021-01492-x)
Supplement: Supplementary file 1 — Additional file 1: Figure S1. OS of patients with HER2-low tumors, patients with completely HER2-negative tumors (HER2-0) and patients with HER2-positive tumors (HER2-pos) in the overall population (n = 1,729). Figure S2. OS of patients with HER2-low tumors and patients with completely HER2-negative tumors (HER2-0) in the overall population (n = 1,378). Figure S3. PFS of patients with HER2-low tumors and patients with completely HER2-negative tumors (HER2-0) in the HR+ population (n = 961). Figure S4. PFS of patients with HER2-low tumors and patients with completely HER2-negative tumors (HER2-0) in the HR-negative population (n = 272). Figure S5. OS of patients with HER2 2+ tumors and patients with HER2 0 or 1+ tumors in the overall population (n = 1,378). Figure S6. OS of patients with HER2 2+ tumors and patients with HER2 0 or 1+ tumors in the HR+ population (n = 1,058). Figure S7. OS of patients with HER2 2+ tumors and patients with HER2 0 or 1+ tumors in the HR-negative population (n = 320). Table S1. Multivariate analysis (Cox proportional hazard model) of PFS for HR+ MBC. Table S2. Multivariate analysis (Cox proportional hazard model) of PFS for HR-negative MBC. Table S3. Multivariate analysis (Cox proportional hazard model) of OS for premenopausal patients with HR+ MBC. Table S4. Multivariate analysis (Cox proportional hazard model) of OS for postmenopausal patients with HR+ MBC. Table S5. HR+ model stability investigations according Heinze G. et al. [17]. Table S6. HR+ model selection frequencies according Heinze G. et al. [17]. Table S7. HR- Model stability investigations according Heinze G. et al. [17]. Table S8. HR- Model selection frequencies according Heinze G. et al. [17]. [file 13058_2021_1492_MOESM1_ESM.docx]

Landscape of HER2-low metastatic breast cancer (MBC): results from the AGMT_MBC-Registry.

**Additional file 1**


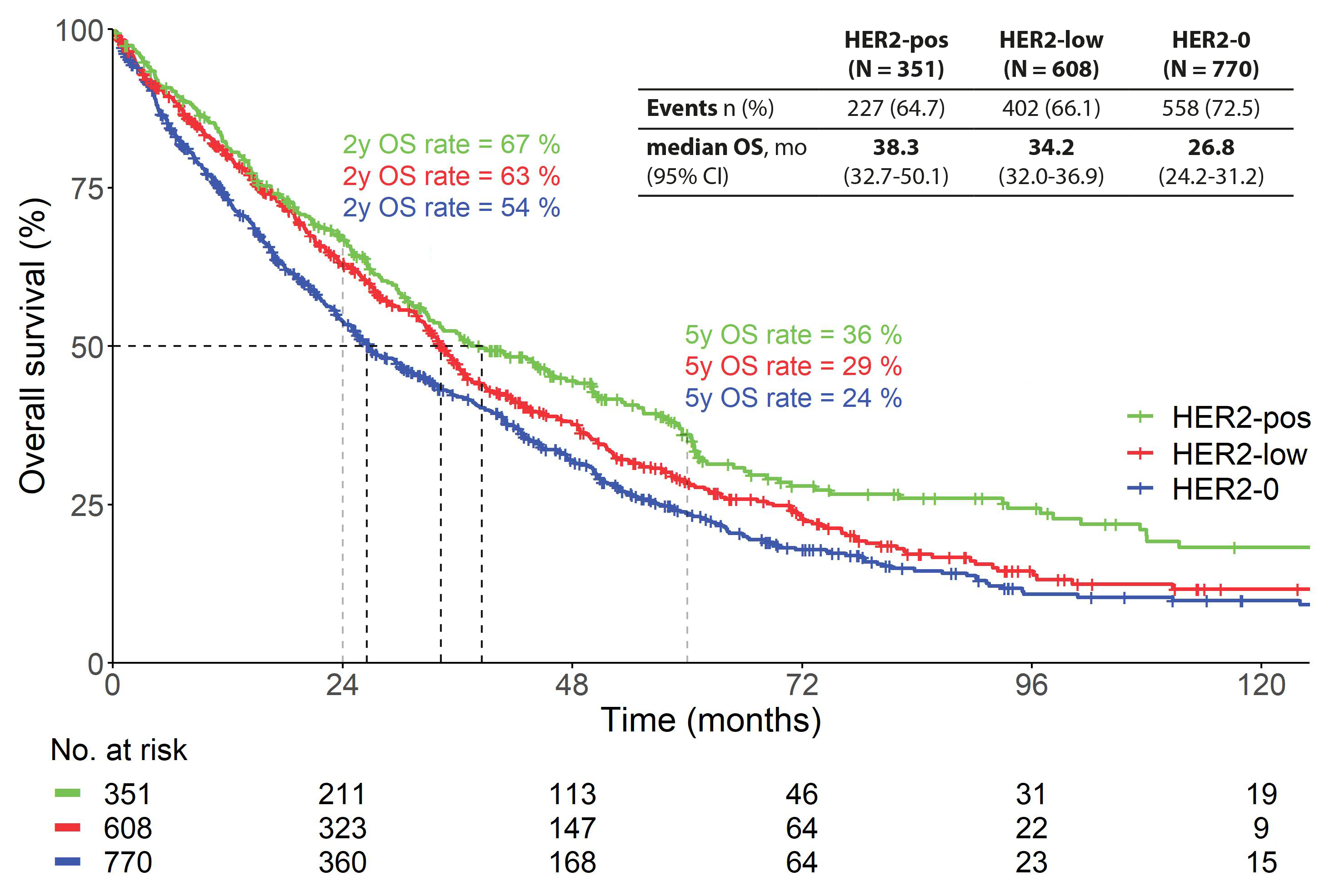


Additional file 1: Figure S1. OS of patients with HER2-low tumors, patients with completely HER2-negative tumors (HER2-0) and patients with HER2-positive tumors (HER2-pos) in the overall population (n = 1,729)


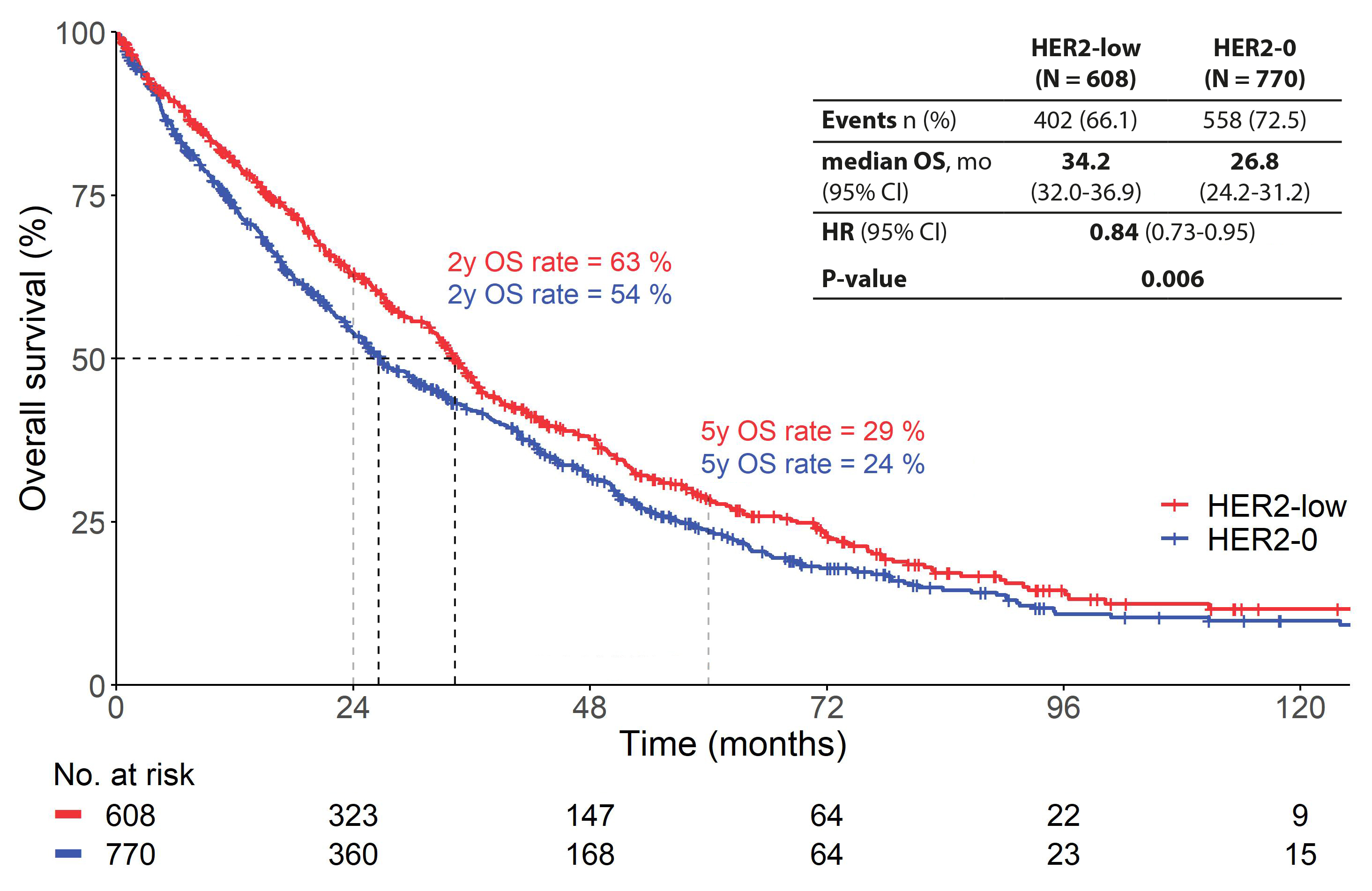


Additional file 1: Figure S2. OS of patients with HER2-low tumors and patients with completely HER2-negative tumors (HER2-0) in the overall population (n = 1,378)


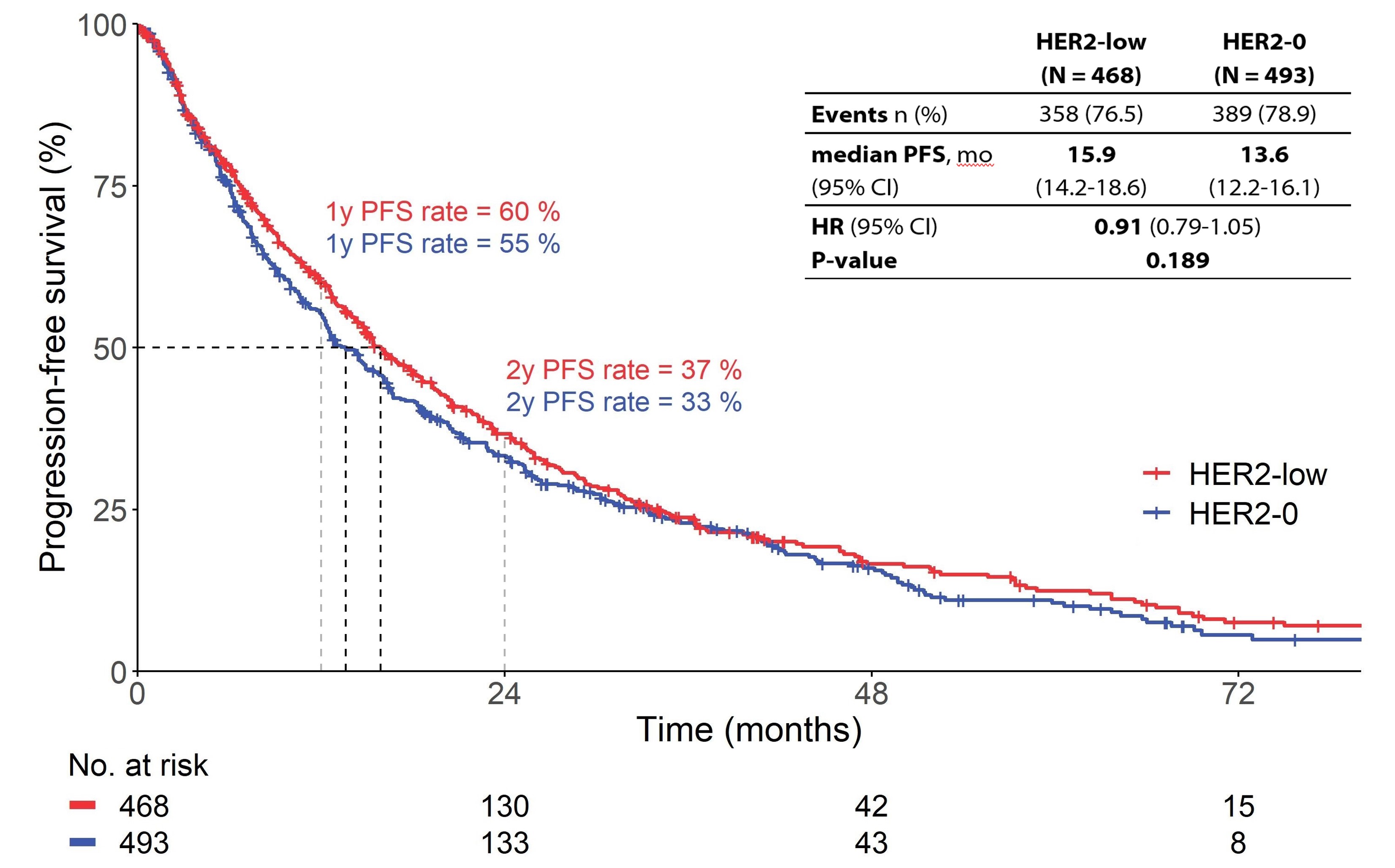


Additional file 1: Figure S3. PFS of patients with HER2-low tumors and patients with completely HER2-negative tumors (HER2-0) in the HR+ population (n = 961).


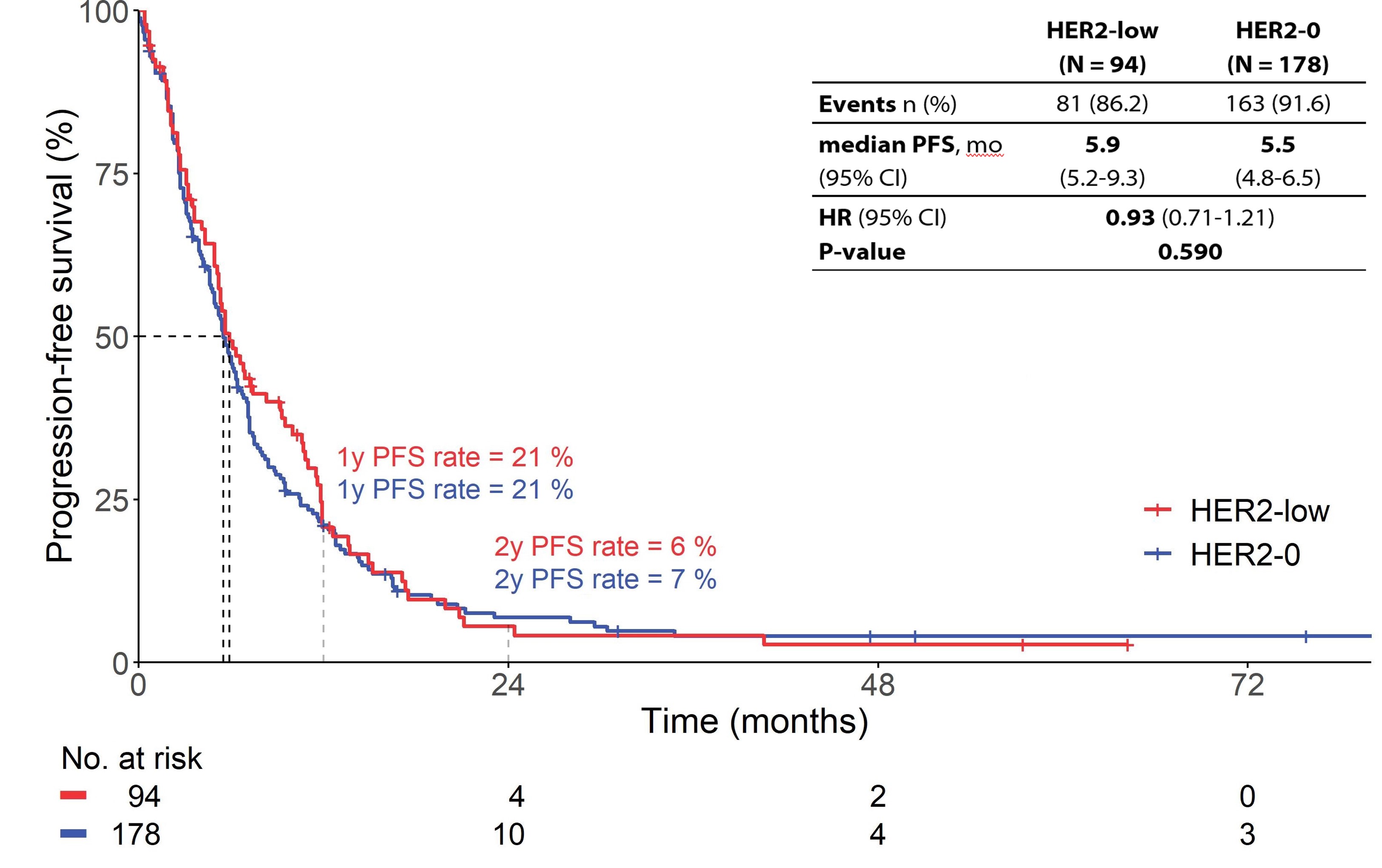


Additional file 1: Figure S4. PFS of patients with HER2-low tumors and patients with completely HER2-negative tumors (HER2-0) in the HR-negative population (n = 272).


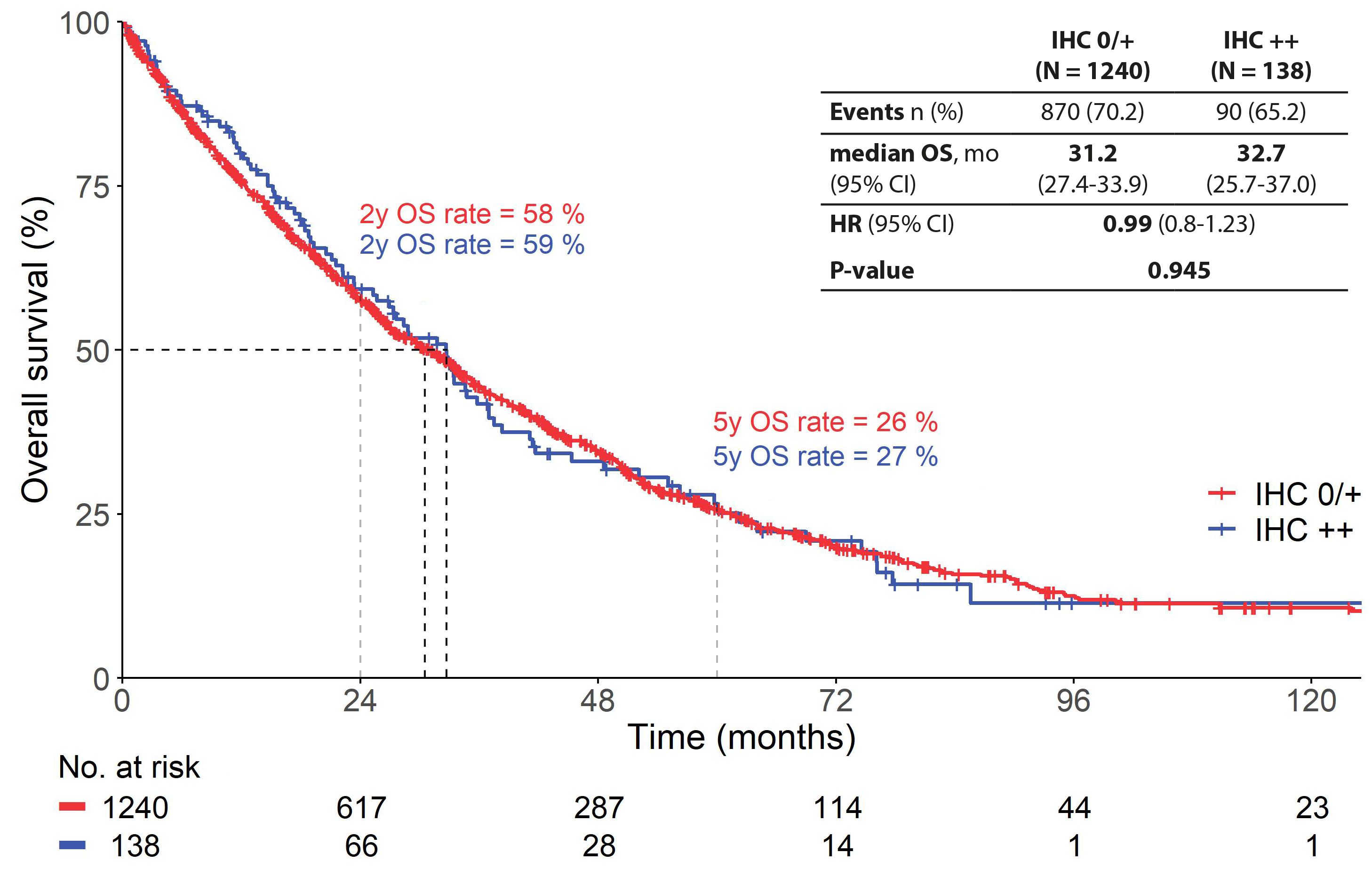


Additional file 1: Figure S5. OS of patients with HER2 2+ tumors and patients with HER2 0 or 1+ tumors in the overall population (n = 1,378)


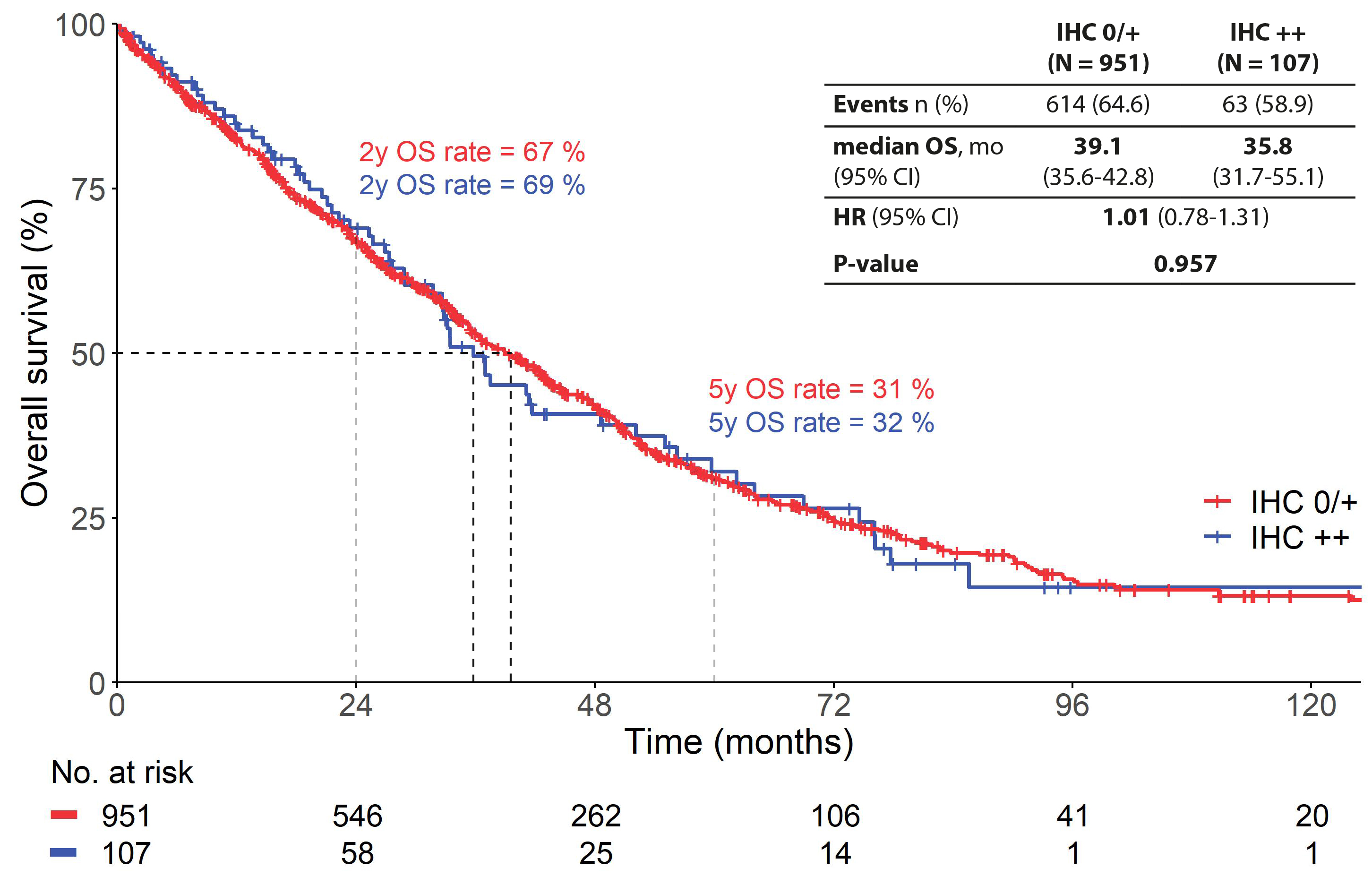


**Additional file 1: Figure S6.** OS of patients with HER2 2+ tumors and patients with HER2 0 or 1+ tumors in the HR+ population (n = 1,058).


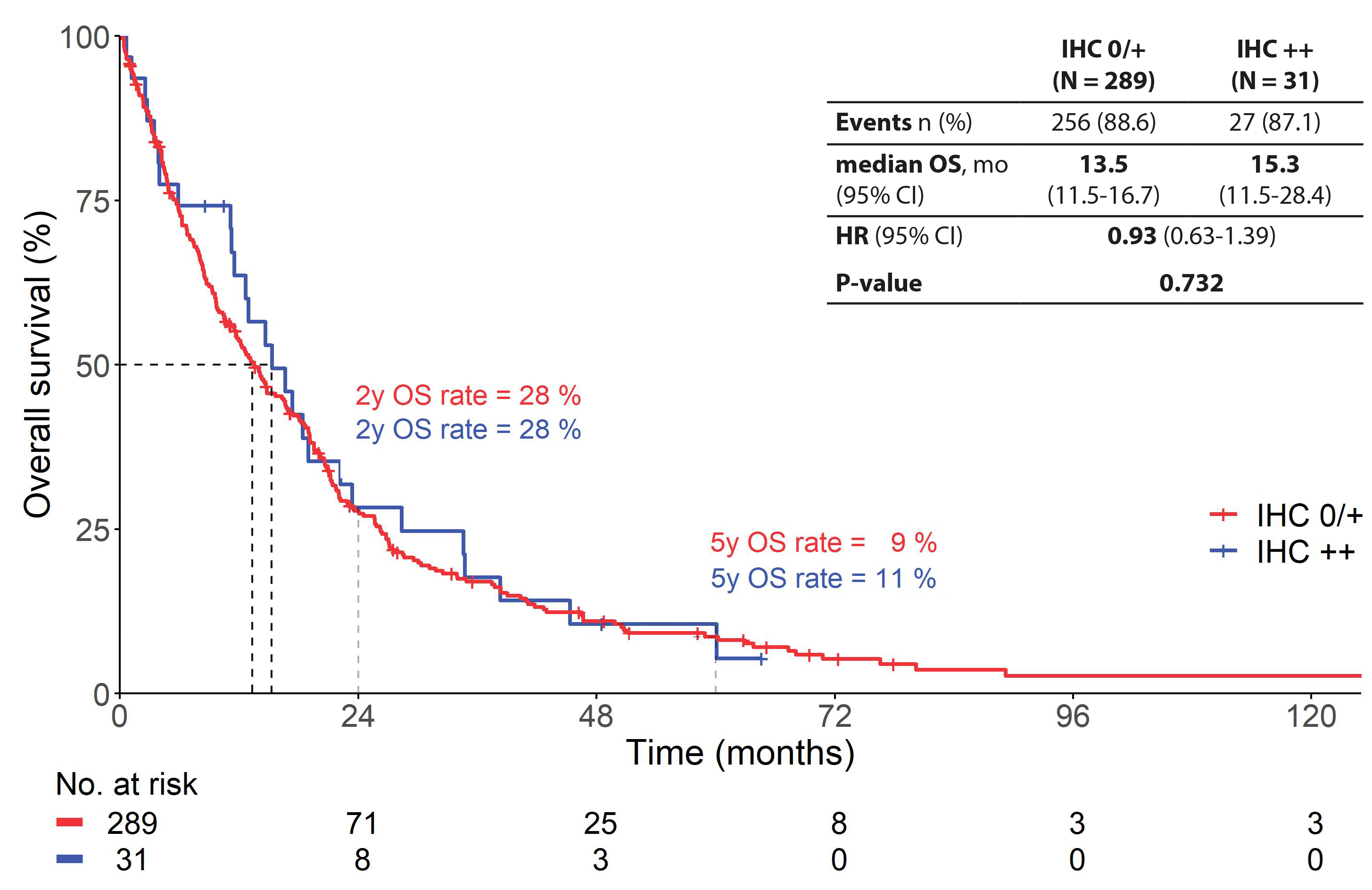


**Additional file 1: Figure S7.** OS of patients with HER2 2+ tumors and patients with HER2 0 or 1+ tumors in the HR-negative population (n = 320).

Additional file 1: Table S1. Multivariate analysis (Cox proportional hazard model) of PFS for HR+ MBC.

| **N = 754** (events 567) | **HR** | **95%CI** | ***P*** |
| --- | --- | --- | --- |
| **Age** (continuous) **according to menopausal status**  Premenopausal  Postmenopausal | 1.01  1.01 | 1.00 - 1.03  1.00 - 1.02 | 0.128  0.055 |
| **DFS**  *≥ 24 months or de novo vs. < 24 months* | 0.71 | 0.56 – 0.91 | **0.008** |
| **Visceral vs. no visceral disease*** | 1.16 | 0.95 – 1.41 | 0.152 |
| **Number of metastatic sites***  *2-3 vs. 1 ≥ 4 vs. 1* | 1.24  1.36 | 1.02 - 1.51  0.90 – 2.05 | **0.029**  0.147 |
| **HER2-low vs. HER2-0** | 0.92 | 0.78 – 1.08 | 0.308 |
| * at diagnosis of metastatic disease | | | |

**Additional file 1: Table S2.** Multivariate analysis (Cox proportional hazard model) of PFS for HR-negative MBC.

| **N = 189** (events 167) | **HR** | **95%CI** | ***P*** |
| --- | --- | --- | --- |
| **Age** (continuous) **according to menopausal status**  Premenopausal  Postmenopausal | 1.03  1.01 | 1.00 - 1.05  1.00 - 1.03 | **0.033**  0.122 |
| **DFS**  *≥ 24 months or de novo vs. < 24 months* | 0.60 | 0.43 – 0.83 | **0.002** |
| **Visceral vs. no visceral disease*** | 1.33 | 0.95 – 1.85 | 0.095 |
| **Number of metastatic sites***  *2-3 vs. 1 ≥ 4 vs. 1* | 1.35  1.54 | 0.96 – 1.91  0.84 – 2.82 | 0.084  0.163 |
| **HER2-low vs. HER2-0** | 0.98 | 0.70 – 1.37 | 0.908 |
| * at diagnosis of metastatic disease | | | |

**Additional file 1: Table S3.** Multivariate analysis (Cox proportional hazard model) of OS for premenopausal patients with HR+ MBC.

| **N = 113** (events 75) | **HR** | **95%CI** | ***P*** |
| --- | --- | --- | --- |
| **Age** (continuous) | 1.01 | 0.98 - 1.04 | 0.620 |
| **DFS**  *≥ 24 months or de novo vs. < 24 months* | 0.50 | 0.30 – 0.84 | **0.009** |
| **Visceral vs. no visceral disease*** | 2.00 | 1.11 – 3.61 | **0.021** |
| **Number of metastatic sites***  *2-3 vs. 1 ≥ 4 vs. 1* | 1.03  2.00 | 0.57 - 1.87  0.64 – 6.25 | 0.920  0.232 |
| **HER2-low vs. HER2-0** | 1.10 | 0.67 – 1.82 | 0.705 |
| * at diagnosis of metastatic disease | | | |

**Additional file 1: Table S4.** Multivariate analysis (Cox proportional hazard model) of OS for postmenopausal patients with HR+ MBC.

| **N = 719** (events 450) | **HR** | **95%CI** | ***P*** |
| --- | --- | --- | --- |
| **Age** (continuous) | 1.04 | 1.03 - 1.05 | **<0.001** |
| **DFS**  *≥ 24 months or de novo vs. < 24 months* | 0.91 | 0.69 – 1.21 | 0.529 |
| **Visceral vs. no visceral disease*** | 1.16 | 0.93 – 1.45 | 0.185 |
| **Number of metastatic sites***  *2-3 vs. 1 ≥ 4 vs. 1* | 1.27  1.72 | 1.02 - 1.58  1.12 – 2.65 | **0.031**  **0.013** |
| **HER2-low vs. HER2-0** | 0.84 | 0.70 – 1.01 | 0.069 |
| * at diagnosis of metastatic disease | | | |

**Additional file 1: Table S5.** HR+ model stability investigations according Heinze G. et al. [17].

|  | **Global model** |  | **Selected model** |  |  |  | |
| --- | --- | --- | --- | --- | --- | --- | --- |
| **Predictors** | **HR (Std. Error)** | **Bootstrap inclusion frequency (%)** | **Estimate (Std. Error)** | **RMSD ratio** | **Relative cond. bias (%)** | **Bootstrap median (boot. 95%-CI)** | |
| **Age** (continuous) **according to menopausal status**  Premenopausal  Postmenopausal | 1.04 (1.00)  1.03 (1.00) | 100 | 1.04 (1.00)  1.03 (1.00) | 0.01  0.01 | 0.08  0.04 | 1.04 (1.03-1.06)  1.03 (1.02-1.04) | |
| **DFS**  *≥ 24 months or de novo vs. < 24 months* | 0.75 (1.01) | 100 (fixed) | 0.75 (1.01) | 0.11 | 1.27 | 0.75 (0.56-1.02) | |
| **Visceral vs. no visceral disease*** | 1.26 (1.01) | 100 (fixed) | 1.26 (1.01) | 0.16 | 1.85 | 1.27 (1.01-1.65) | |
| **Number of metastatic sites***  *2-3 vs. 1 ≥ 4 vs. 1* | 1.25 (1.01)  1.73 (1.02) | 87  87 | 1.25 (1.01)  1.73 (1.02) | 0.47  0.74 | 2.66  10.10 | 1.25 (0.00-1.54)  1.77 (0.00-2.84) | |
| **HER2-low vs. HER2-0** | 0.89 (1.00) | 100 (fixed) | 0.89 (1.00) | 0.08 | 1.05 | 0.89 (0.74-1.06) | |
| * at diagnosis of metastatic disease | | | | | | |  |

**Additional file 1: Table S6.** HR+ model selection frequencies according Heinze G. et al. [17].

| **Model** | **Included predictors** | **Count** | ***Percent*** | ***Cumulative percent*** |
| --- | --- | --- | --- | --- |
| 1 | Age:menopausal status, DFS, Visceral disease, Nr. of metastatic sites, HER2-low/0 | 870 | 87 | 87 |
| 2 | Age:menopausal status, DFS, Visceral disease, HER2-low/0 | 130 | 13 | 100 |

**Additional file 1: Table S7.** HR- Model stability investigations according Heinze G. et al. [17].

|  | **Global model** |  | **Selected model** |  |  |  |
| --- | --- | --- | --- | --- | --- | --- |
| **Predictors** | **HR (Std. Error)** | **Bootstrap inclusion frequency (%)** | **Estimate (Std. Error)** | **RMSD ratio** | **Relative cond. bias (%)** | **Bootstrap median (boot. 95%-CI)** |
| **Age** (continuous) **according to menopausal status**  Premenopausal  Postmenopausal | 1.04 (1.00)  1.02 (1.00) | 91.6 | 1.04 (1.00)  1.02 (1.00) | 0.30  0.30 | 0.39  0.29 | 1.04 (0.00-1.07)  1.03 (0.00-1.04) |
| **DFS**  *≥ 24 months or de novo vs. < 24 months* | 0.59 (1.01) | 100 (fixed) | 0.59 (1.01) | 0.09 | 1.75 | 0.60 (0.44-0.80) |
| **Visceral vs. no visceral disease*** | 1.59 (1.01) | 100 (fixed) | 1.59 (1.01) | 0.32 | 6.88 | 1.67 (1.20-2.38) |
| **Number of metastatic sites***  *2-3 vs. 1 ≥ 4 vs. 1* | 1.23 (1.01)  2.01 (1.04) | 73.7  73.7 | 1.23 (1.01)  2.01 (1.04) | 0.65  1.44 | 5.77  30.52 | 1.18 (0.00-1.73)  2.05 (0.00-4.88) |
| **HER2-low vs. HER2-0** | 0.92 (1.01) | 100 (fixed) | 0.92 (1.01) | 0.16 | -0.31 | 0.91 (0.65-1.26) |
| * at diagnosis of metastatic disease | | | | | | |

**Additional file 1: Table S8.** HR- Model selection frequencies according Heinze G. et al. [17]

| **Model** | **Included predictors** | **Count** | ***Percent*** | ***Cumulative percent*** |
| --- | --- | --- | --- | --- |
| 1 | Age:menopausal status, DFS, Visceral disease, Nr. of metastatic sites, HER2-low/0 | 663 | 66.3 | 66.3 |
| 2 | Age:menopausal status, DFS, Visceral disease, HER2-low/0 | 253 | 25.3 | 91.6 |
| 3 | DFS, Visceral disease, Nr. of metastatic sites, HER2-low/0 | 74 | 7.4 | 99.0 |
| 4 | DFS, Visceral disease, HER2-low/0 | 10 | 1.0 | 100 |
